# The Metabonomic Studies of Tongue Coating in *H. pylori* Positive Chronic Gastritis Patients

**DOI:** 10.1155/2015/804085

**Published:** 2015-10-19

**Authors:** Xuan Liu, Zhu-Mei Sun, Yan-Na Liu, Qing Ji, Hua Sui, Li-Hong Zhou, Fu-Feng Li, Qi Li

**Affiliations:** ^1^Department of Medical Oncology, Shuguang Hospital, Shanghai University of Traditional Chinese Medicine, Shanghai 201203, China; ^2^Shanghai University of Traditional Chinese Medicine, Shanghai 201203, China

## Abstract

In Traditional Chinese Medicine (TCM), tongue diagnosis (TD) has been an important diagnostic method for the last 3000 years. Tongue coating can be used as a very sensitive marker to determine the progress of chronic gastritis. Therefore, the scientific, qualitative, and quantitative study for the pathophysiologic basis of tongue coating (TC) emerged as a major direction for the objective research of TD. In our current report, we used GC/MS technology to determine the potential changes of metabolites and identify special metabolic biomarkers in the TC of *H. pylori* infected chronic gastritis patients. Four discriminative metabolites were identified by GC/MS between the TC of *H. pylori* infection (G + H) and without *H. pylori* infection (G − H) patients: ethylene, cephaloridine, *γ*-aminobutyric acid, and 5-pyroglutamic acid, indicating that changes in amino acid metabolism are possibly involved in the formation of TC, and the amino acid metabolites are part of the material components of TC in G + H patients.

## 1. Introduction

*Helicobacter pylori* (*H. pylori*,* Hp*) infection is one of the most important causes of chronic gastritis and gastric cancer [1, 2].* Hp*, a Gram-negative bacterium found in the stomach, is listed as Class I carcinogen by WHO. In 1984,* Hp* was first isolated from the gastric mucosa and epithelial surface by Marshall and Warren [3].* Hp* infection can lead to chronic gastritis, gastric and duodenal ulcers, and increased risk of gastric cancer [1, 4–7]. Correa delineated the whole pathological process from* Hp* infection induced inflammation of gastric mucosa, to intestinal metaplasia, aplasia, and carcinoma [8]. In 1998, Watanabe et al. established the first animal model with Mongolian gerbils to demonstrate that* Hp* infection directly causes gastric cancer [9]. Our previous studies illustrated that* Hp* can grow in the stomach mucosa of C57BL/6 mice following oral gavage of the bacteria. Seventy-two weeks later, pathological examinations clearly revealed a 22.2% rate of gastric cancer incidence in the mice [10]. This piece of evidence again reaffirms the role of* Hp* in inducing gastric cancer; however, the mechanism remains elusive, is thought to be very complex, and involved numerous metabolic pathways in the body. This poses a huge challenge for the prevention and treatment of* Hp* induced chronic gastritis and gastric cancer. Among the many current studies of metabolic pathways and the metabolites in* Hp* induced chronic gastritis and cancer, Shi revealed that, in the serum samples of* Hp* infected patients, the activity of superoxide dismutase (SOD) and glutathione peroxidase (GSH-Px) is significantly lower while the malonaldehyde (MDA) level is higher relative to* Hp* negative patients [11].

Tongue diagnosis is a noninvasive, simple, and valuable diagnostic tool, the use of which has been repeatedly affirmed by clinical practitioners of traditional Chinese medicine (TCM) for 3,000 years. Tongue appearance is closely associated with the physiology as well as pathophysiology of the digestive system [12–14]. TCM theories state that the tongue coating (TC) is condensed stomach “Qi” and the essence “Qi” of food; tongue appearance is a very sensitive index of the physiological and pathological status of the organs, especially the stomach and spleen. Tongue appearance reflects the amount of bad “Qi” and the dynamic process of illness of the stomach, that is, the* Hp* infection status. Clinical research has reported that the tongue appearance changes provide essential information for the diagnosis, treatment, and prognosis of chronic gastritis, peptic ulcers, and gastric and colorectal cancers [15–18]. Huang et al. discovered that, in the patients with* Hp* induced chronic superficial gastritis, the colors of TC were mainly light white and yellow [19]. This finding was corroborated by another report by Wang et al., which suggests that, in 518 chronic gastritic cases, 81.6% of* Hp*-infection positive patients had yellow CT, significantly higher than the* Hp* negative group [20]. Mao reported that the tongue color of the majority of* Hp* positive patients was light red while the TC appeared greasy, thick, and yellow [21]. Together, these and other studies have demonstrated that tongue appearance correlates with* Hp* infection status: positive patients have red or purple tongues with yellow TC, and the more severe the* Hp* infection gets, the thicker and greaser the TC appears. Tongue appearance also reflects the degrees of gastric inflammation and prognosis: when TC turns to be thinner, it indicates a better function status of stomach and spleen, less inflammation, and less* Hp* infection [22].

Metabonomics is an important part of the system biology. Metabolites are the ultimate products of gene expression, closely related to the physiology and pathophysiology of the body. Metabonomics considers the human body as a whole system, which is consistent with the TCM concept, and therefore has wide application prospects in TCM research [23–25]. The analysis of syndrome (“zheng” in TCM) associated metabolites may help comprehend the changes of metabolic pathways and conditions when a disease progresses and understand the material basis of the disease. Chen et al. reported a specific metabolite, 1-methyladenosine, as biomarker in hepatocellular carcinoma patients using metabonomics [26]; Leichtle et al. investigated the levels of 26 amino acids in the blood of colorectal cancer patients and found that the cancer patients had lower concentration for 11 amino acids and proposed a carcinoembryonic antigen- (CEA-) glycine-tyrosine tri-biomarker, the best model for the diagnosis of the disease [27]. Chronic gastritis is associated with* Hp* infection and TC is a reliable status indicator of* Hp* infection, gastric inflammation, and prognosis. Hence, in the current study, we used GC/MS technology to investigate the spectrum of material composition in TC of* Hp* infected patients, determine the changes of TC metabolites, and identify microorganism biomarkers for the* Hp* positive, chronic gastritis patients.

## 2. Materials and Methods

### 2.1. Ethical Statement

All samples were obtained as part of diagnostic criteria after patients gave written informed consent. The study was approved by the local ethics committee of Shanghai University of Traditional Chinese Medicine Shuguang Hospital (SUTCMSH), Shanghai, China.

### 2.2. Participant Selection Criteria and TC Samples Details

The participants of this study were mainly patients from SUTCMSH, from October 2012 to July 2013. All patients underwent a gastroscopy examination for diagnosis of chronic gastritis and a gastric mucosa biopsy and Giemsa staining to confirm* Hp* infection. Twenty-nine patients had both chronic gastritis and* Hp* infection while 13 patients had only chronic gastritis. Our study included 42 chronic gastritis patients at Shuguang Hospital, Shanghai University of Traditional Chinese Medicine, between October 2012 and July 2013. Of the 42 cases, 23 were positive of* Hp* infection (12 male, 11 female), with the mean age of 51.71 ± 13.42 years; 19 were negative of* Hp* infection (7 males, 12 females), with the mean age of 58.57 ± 10.69 years. The TC color in the* Hp* group was mainly yellow while mainly white or white/yellow in the non-*Hp* group (Table 1). No significant differences in gender and age were observed between the two groups (*P* > 0.05).

### 2.3. Tongue Coating (TC) Samples Collection

**Figure 1:**
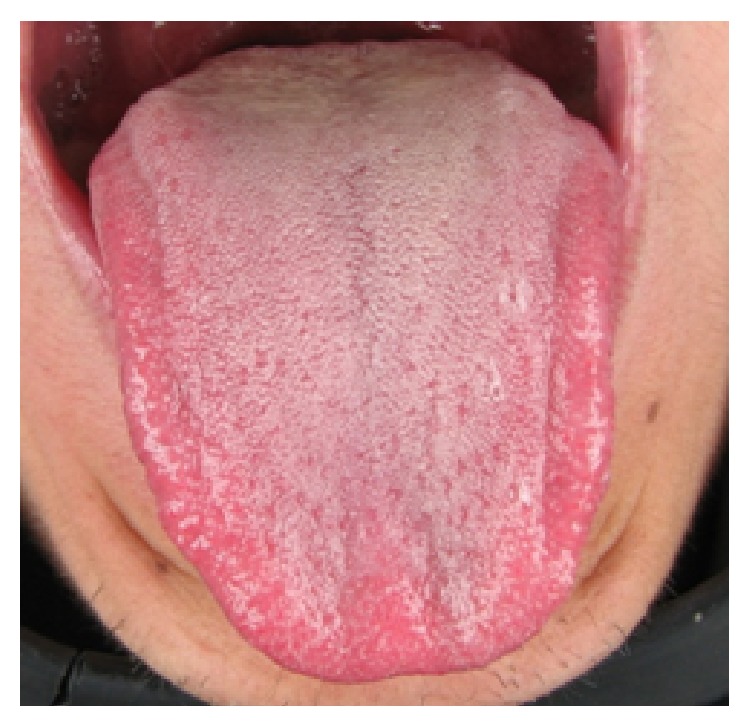
Sampling images of tongue coating from the center of the tongue, an area regarded as tongue coating in the traditional tongue diagnosis.

The TC samples were collected as previously described [22]. All participants were required to gargle saline 2~3 times before sampling to rinse possible food contamination that might influence the TC. Small spoons were used to scrape the TC at the thickest area and samples were placed into sanitized Eppendorf tubes that had been filled with 2 mL of sterile saline. All samples were stored at −80°C until analysis (Figure 1).

### 2.4. Gas Chromatography-Mass Spectrometry (GC/MS) Analysis

#### 2.4.1. GC/MS Measurement

TC samples were prepared by sonication and centrifugation at 4°C 3500 rpm for 10 min. The 100 *μ*L supernatant was transferred to a new tube and after adding 200 *μ*L methanol, it was vortexed for 30 s, incubated at −20°C for 10 min, and centrifuged at 4°C 10000 rpm for 10 min; then 200 *μ*L supernatant was transferred to a sample tube. Sample was then freeze-dried, added 10 *μ*L of chlorophenylalanine (0.3 mg/mL) and 30 *μ*L of methoxamine pyridine (15 mg/mL), sealed, vortexed for 30 s, incubated at 37°C for 90 min, added 40 *μ*L BSFTA (containing 1% TMCS), incubated at 80°C for 2 h, cooled at room temperature for 1 h, before being analyzed by GC/MS. The GC/MS system was from Agilent Technology (California, USA), Model# DB5MS, column: 30 m × 0.25 mm × 0.25 *μ*m. GC/MS condition is as follows: the column temperature was held at 80°C for 3 min, then 10°C/min increased to 140°C, 4°C/min increased to 240°C, 10°C/min increased to 280°C, and it was held for 10 min. Injection inlet temperature was 280°C and sensor temperature was 300°C. The carrier gas is Helium and flow rate was 1 mL/min. MS condition is as follows: EI ionization, electron energy 70 eV, ion source temperature 250°C, interface temperature 250°C, solvent delay 5 min, full-spectral scan, and scan scope M/Z 40–600.

#### 2.4.2. GS/MS Data Analysis

Raw data was processed through multiple stages including noise reduction, feature detection, alignment of peaks, and normalization. GC/MS data were analyzed by Agilent Mass Profiler Professional (MPP) software. We analyzed the previously processed data by SIMCA-P^+^ software (V13.0, Umetrics AB, Umea, Sweden), principal component analysis (PCA) is used to analyze the data by Centered Scaling method, and the data is automatically modeled and analyzed; partial least squares-discriminate analysis (PLS-DA) is used to analyze the data by Centered Scaling method, and the data is automatically modeled, modeling analysis of the first and two principal components; and orthogonal partial least squares-discriminate analysis (OPLS–DA) showed the maximum differences between different groups within the model.

#### 2.4.3. Identification of Metabolite Markers

Differential metabolites markers were selected according to the PLS-DA Variable Importance in the Projection (VIP), considering only variables with VIP values higher than 1, indicative of significant differences among groups. These potential markers were identified by retention time correction of peaks and mass-to-charge ratio (*m/z*) using the Mass Spectral Library (National Institute of Standards and Technology, NIST).

## 3. Results

### 3.1. Chromatographic Analysis and Comparison between the* Hp*-Infection Positive and Negative Chronic Gastritis Patients

**Figure 2:**
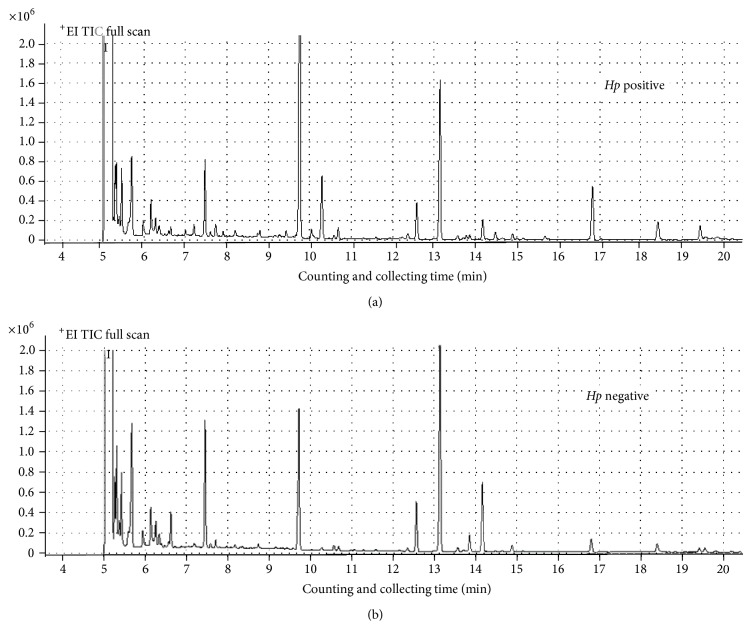
GC/MS metabolic fingerprinting total ion chromatogram of TC samples from* Hp* positive and* Hp* negative chronic gastritis patients.

The total ion chromatograms obtained by GC/MS from the TC samples of* Hp* positive and* Hp* negative chronic gastritis patients demonstrated a clear difference between the two groups (Figure 2). In order to determine the detailed metabolomic profiles, multivariate statistical analysis was performed for the samples, that is, the principal component analysis (PCA), partial least squares-discriminant analysis (PLS-DA), and orthogonal partial least squares-discriminate analysis (OPLS-DA).

### 3.2. The Metabonomics of TC Samples from the* Hp* Positive and* Hp* Negative Chronic Gastritis Patients

**Figure 3:**
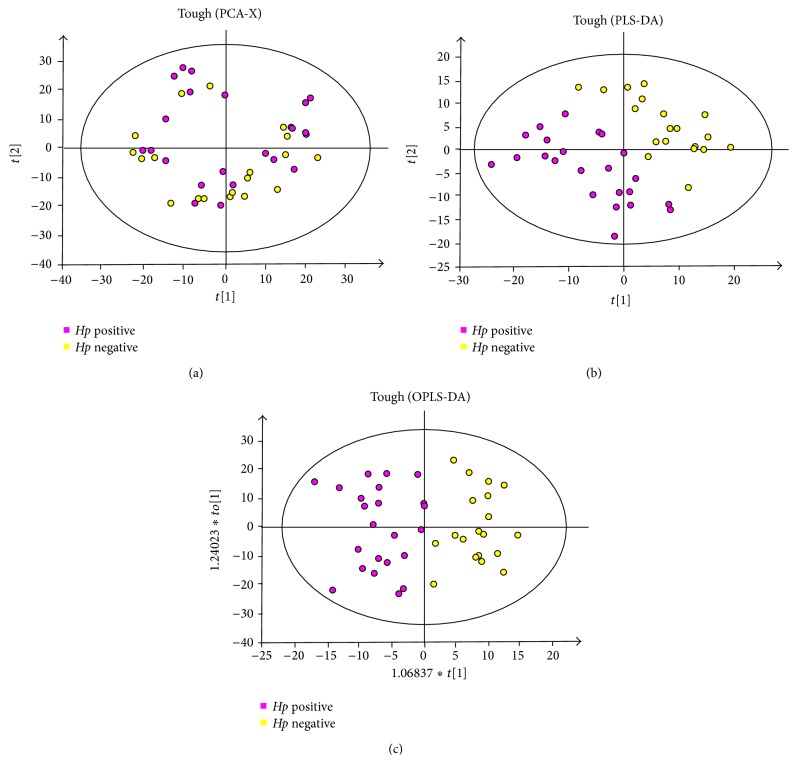
The metabonomics of tongue coating samples from* Hp* positive and* Hp* negative chronic gastritis patients. (a) PCA analysis, (b) PLS-DA analysis, and (c) OPLS-DA analysis.

PCA scores of the TC samples from the two groups showed that all the sample points fell in the 95% confidence intervals but appeared partially overlapped, indicating that this method was not able to discriminate between the groups (Figure 3(a)). Further research by PLS-DA showed that the sample points were clearly separated (obtaining good class separation value and predictive power, with *R*_2_ *Y* = 0.82), which indicated that the two groups' metabolic pathways were different: all the sample points of* Hp* positive patients were mainly in the left lower quadrant, while the* Hp* negative patients' sample points are in the right upper quadrant (Figure 3(b)). To improve the accuracy of the PLS discriminated model, OPLS–DA by SIMCA-P^+^ software was used to analyze the results to better highlight the difference between the groups. The analysis result showed that the sample points from the two groups were completely separated in different quadrants: the* Hp* positive sample points were in the left quadrant and* Hp* negative in the right (Figure 3(c)).

### 3.3. The Different Metabolite Markers of TC Samples from the* Hp* Positive and* Hp* Negative Chronic Gastritis Patients

We used OPLS-DA to block out irrelevant signals, to acquire reliable metabolite marker peaks. The metabolites responsible for discrimination were selected according to the Variable Importance in the Projection (VIP) considering only variables with VIP values higher than 1.0, indicative of significant differences among groups (Table 2). These potential metabolite markers, identified using the NIST Mass Spectral Library and KEGG bioinformatics database, were *ϒ*-aminobutyric acid, 5-hydroxyproline, ethylene, and some amino acids (Table 3).

## 4. Discussion

As a unique method, the tongue diagnosis contributed a great deal for the formation and development of TCM theory system [28]. “Huang Di Nei Jing,” an ancient TCM book written in Qin and Han era (~2000 years ago), recorded the uses of tongue diagnosis. A chapter in that book called “Ling Shu, Shi Zhuan” stated the following: “By observing lip and tongue, one can determine the stages of a disease.” Tongue coating (TC), as the main part of the tongue appearance, is the moss or fur like material on the tongue surface. The TCM believes that the changes of TC reflect human body's physiology and pathophysiology status. As described by “Xing Se Jian Mo”: “the TC is formed by stomach (“stomach-Qi” in Chinese) and the five organs (“Wu-Zang” in Chinese) are all supplied by the stomach, so the TC is the index of body status.” “New Ling Shu” explained: “Tongue is closely related to the digestive system, whenever the digestive organs have problem, TC will show it.” Therefore, not only can TC be an indicator of the pathophysiological status of the five essential organs, but also a “window” for the development stages of the gastric illnesses, a sensitive index for the progression of chronic gastritis [29]. In summary, to investigate the underlying mechanisms of TC formation, we can explore the nature of chronic gastritis TCM syndromes and obtain new clues and novel ideas for the objective studies of TCM syndromes.

*Hp* infection, one of the most causative factors of chronic gastritis and gastric cancer, has been listed as Class I carcinogen by WHO cancer institutions. Wang et al. reported that, in 518 chronic gastritis patients, 440 cases were* Hp*-infection positive (85%) and mainly had yellow TC (81.16%), significantly higher than the* Hp* negative group [20]. The reason for the yellow appearance of the TC was probably due to* Hp* infection increased gastric inflammation, which led to the malfunctioning of digestive system, lowered saliva secretion, and decreased oral cavity self-cleaning. This resulted in tongue surface dysbacteriosis that caused inflammation, exudate, and yellow-color change of the tongue. This is just a hypothesis, which apparently needs to be studied further and supported by experimental evidence. Therefore, how the* Hp* infection causes TC changes still remains an unsolved problem.

Metabolomics, based on the analysis of the entire set of metabolites in a sample, provides a comprehensive overview of the status of organisms, more directly and accurately reflecting the pathophysiology of the organisms. Biomarkers discovery is the current research “hotspot,” but most of the metabolite biomarkers are identified in blood, urine, and tissue samples, rarely in TC samples. TC metabolomics, the study of the metabolites of TC samples to determine the pathophysiology status of the human body, has recently emerged. Li et al. established the methodology to process TC sample for metabolomics analysis [30]. Sun et al. discovered 10 discriminative metabolite biomarkers between TC samples of normal and chronic gastritis groups, using LC/MS technology [25]. Zhao et al. utilized GC/MS technology to uncover 17 metabolite biomarkers between normal and chronic hepatitis groups [31]. TC, as biological sample, is convenient and noninvasive to collect and is unique to TCM, which believes that TC is condensed Qi and liquid (“Jin” in Chinese) evaporated from the spleen and stomach on the tongue surface, so TC reflects the physiological and pathological status of the human body. Our current report researched TC samples to determine metabolite biomarkers in the TC of the* Hp* infection induced chronic gastritis patients.

We used PLS-DA and OPLS-DA statistical methods to analyze the GC/MS data of TC samples from* Hp* positive and* Hp* negative chronic gastritis patients and found a difference between the metabolites of each group. Using the NIST Mass Spectral Library and KEGG bioinformatics database, we identified these discriminative metabolite biomarkers as *ϒ*-aminobutyric acid, 5-hydroxyproline, ethylene, and pyroglutamic acid which is derived from glutamine through dehydration and cyclization. Glutamine is one of the 20 common amino acids of the human body. It can form glutathione (GSH) by synthetically reacting with cysteine and glycine [32]. GSH plays a role in the biodefense system of the human body, that is, proimmunity, antiaging, and detoxicating. The TC samples of the* Hp* positive chronic gastritis patients had higher amount of pyroglutamic acid (VIP > 1), which indicates that the synthesis pathway of GSH was blocked, as the glutamine was not used to make GSH but directed toward the dehydration/cyclization reaction, to form pyroglutamic acid. The imbalance of GSH metabolism will disrupt normal physiology, causing a decrease of immune and detoxicating functions of the human body. McNulty and Dent uncovered that highly homogeneous groups of* C. pylori* produce a similar panel of enzymes, including oxidase, DNase, oxidase, catalase, urease, alkaline phosphatase, leucine aminopeptidase, and *ϒ*-glutamyl aminopeptidase [33]; therefore, our future research projects will be focused on interrogating whether the* Hp* produced *ϒ*-glutamyl aminopeptidase affects the metabolism of glutamine.

## 5. Conclusions

We used GC/MS technology to determine the metabolic components of tongue coating samples in chronic gastritis patients with or without* Hp* infection. We found distinct metabonomic differences between the 2 patient groups and identified 4 discriminative metabolite biomarkers in the tongue coating of* Hp* positive chronic gastritis patients: ethylene, cephaloridine, *γ*-aminobutyric acid, and 5-pyroglutamic acid. The discovery of these metabonomic biomarkers in the tongue coating not only can help the diagnosis and treatment of* Hp* infection induced chronic gastritis, but also provide a theoretical basis for the utilization of tongue coating aided clinical diagnosis of diseases.

## Supplementary Material

In supplement material, we showed the tongue coating different peak areas between Hp positive group (TH) and Hp negative group (TN) by GC/MS in chronic gastritis. In GC/MS +EI TIC full scan, there were many peaks in different retention times, different retention times is correspond to the different peak area.
Var ID: number; Peak: mass@retention time; MEAN TH: mean TH peak area; MEAN TN: mean TN peak area

## Figures and Tables

**Table 1:** General information of the chronic gastric patients.

Group	Tongue coating White/yellow W/Y	Gender		Age ($\bar{x}$ ± SD)
		Male	Female	
*Hp* positive	$\begin{array}{ccc} 2 & 13 & 8 \end{array}$	12	11	51.71 ± 13.42
*Hp* negative	$\begin{array}{ccc} 9 & 1 & 9 \end{array}$	7	12	58.57 ± 10.69

**Table 2:** Comparison of chromatogram peaks from the TC samples of *Hp* positive and *Hp* negative chronic gastritis patients.

Var ID	*m*/*z*	Mean peak area	Mean peak area	*P* value	VIP
		(*Hp* positive)	(*Hp* negative)		
593	73	0.000124052	0.000290037	0.04129	2.0532
64	73	0.000231336	8.16838*E* − 05	0.04928	2.05431
512	73	0.000162672	2.74211*E* − 05	0.03997	2.09457
623	73	7.71475*E* − 05	0.000303819	0.04789	2.09683
42	43	0.001224892	0.000927157	0.05121	2.17178
11	73	0.022093242	0.037576638	0.04812	1.06903
668	73	0.000341666	5.39923*E* − 05	0.05087	2.29584
799	73	0.000312782	0.004340828	0.04879	2.62218
680	73	0.000954827	0.000677735	0.04762	2.75425
595	73	0.000208521	5.79851*E* − 5	0.03584	2.79777
523	73	5.86622*E* − 05	0.000264136	0.04978	2.87228
321	57	0.000112378	9.7488*E* − 05	0.05041	1.16753

Note: we selected the different materials between *Hp* positive and *Hp* negative patients with *P* < 0.05, VIP > 1.0.

**Table 3:** The potential metabolite biomarkers and related metabolic pathways in the TC of *Hp* positive and *Hp* negative chronic gastritis groups.

Var ID	CAS1/NIST	CAS2/NIST	Name	KEGG ID
593	7381-30-8	—	Ethylene	C06547
64	1126-58-5	—	—	—
512	50-59-9	—	Cephaloridine	C11754
623	39508-23-1	—	*γ*-Aminobutyric acid GABA	C00334
42	1126-58-5	—	—	—
668	30274-77-2	—	Pyroglutamic acid	C01879, C02237
799	54477-01-09	55521-23-8	—	—
615	1126-58-5	50-59-9	Cephaloridine	C11754
680	1126-58-5	—	—	—
595	55521-23-8	50-59-9	Cephaloridine	C11754
523	7381-30-8	1126-58-5	Ethylene	C06547
321	39508-23-1	—	*γ*-Aminobutyric acid	C00334

Note: CAS1/NIST is the potential metabolite biomarkers number in NIST Mass Spectral Library, and KEGG ID is the potential metabolite biomarkers number in KEGG bioinformatics database.

## References

[1] Khan M. K., Bemana M. (2012). Association of *Helicobacter pylori* infection and gastric carcinoma. *Mymensingh Medical Journal*.

[2] Konturek P. C., Konturek S. J., Brzozowski T. (2009). *Helicobacter pylori* infection in gastric cancerogenesis. *Journal of Physiology and Pharmacology*.

[3] Marshall B. J., Warren J. R. (1984). Unidentified curved bacilli in the stomach of patients with gastritis and peptic ulceration. *The Lancet*.

[4] Cherdantseva L. A., Potapova O. V., Sharkova T. V., Belyaeva Y. Y., Shkurupiy V. A. (2014). Association of *Helicobacter pylori* and iNOS production by macrophages and lymphocytes in the gastric mucosa in chronic gastritis. *Journal of Immunology Research*.

[5] De Leest H. T., Steen K. S., Bloemena E. (2009). *Helicobacter pylori* eradication in patients on long-term treatment with NSAIDs reduces the severity of gastritis: a randomized controlled trial. *Journal of Clinical Gastroenterology*.

[6] Miyamoto M., Haruma K. (2013). Gastric ulcer and duodenal ulcer. *Nihon Rinsho*.

[7] Wroblewski L. E., Peek R. M., Wilson K. T. (2010). Helicobacter pylori and gastric cancer: factors that modulate disease risk. *Clinical Microbiology Reviews*.

[8] Correa P. (1991). Is gastric carcinoma an infectious disease?. *The New England Journal of Medicine*.

[9] Watanabe T., Tada M., Nagai H., Sasaki S., Nakao M. (1998). *Helicobacter pylori* infection induces gastric cancer in *Mongolian gerbils*. *Gastroenterology*.

[10] Li Q., Liu N., Zhao C. (2010). Establishment of a mouse model of chronic *Helicobacter pylori* infection induced gastric adenocarcinoma and its effect of *Helicobacter pylori* infection on angiogenesis. *World Chinese Journal of Digestology*.

[11] Shi C. (2014). *Chronic atrophic gastritis turbidity toxin nitrinsic card with Hp infection and SOD, MDA, GSH-Px correlation studies [M.S. thesis]*.

[12] Li X. L., Wang Z. D. (2006). Study on the correlation of tongue diagnosis and diseases of spleen and stomach. *Journal of Jiangxi College of Traditional Chinese Medicine*.

[13] Fang H., Ding C., Wang Y. (2013). Tongue significance in syndrome differentiation of chronic atrophic gastritis. *Chinese Journal of Basic Medicine in Traditional Chinese Medicine*.

[14] Shi B., Xu H., Xie J. (2008). Treatise on the significance of chronic gastritis treated with inspection of the tongue in TCM. *Forum on Traditional Chinese Medicine*.

[15] He Y., Hu Z. (2010). Correlation between gastroscopic staging and chromatic quantification of tongue demonstration in patients with peptic ulcer. *Guangdong Medical Journal*.

[16] Dong W., Wu J., Zhang J. (2013). The relationship between tongue fur, serum epidermal growth factor and laboratory parameters in gastric cancer patients. *Journal of Traditional Chinese Medicine*.

[17] Chen Y., Zhu H. (2012). Progress of studies on tongue images in patients with colorectal cancer. *Lishizhen Medicine and Materia Medica Research*.

[18] Jiang B., Liang X., Chen Y. (2012). Integrating next-generation sequencing and traditional tongue diagnosis to determine tongue coating microbiome. *Scientific Reports*.

[19] Huang M., Lin P., Lan S., Zheng J. (2005). Clinical observation on 120 cases of chronic superficial gastritis' picture of the tongue and Hp infection. *Journal of Liaoning College of Traditional Chinese Medicine*.

[20] Wang C., Chen Y., Chen S. (2002). The relationship between *Helicobacter pylori* infection and tongue coating in 518 cases of patients with stomach. *Chinese Journal of Integrated Traditional and Western Medicine*.

[21] Mao Y. (2012). *The correlate research of upper gastrointestinal Helicobacter pylori infection and tongue in TCM [M.D. thesis]*.

[22] Xie J. (2013). *The correlational research on Helicobacter pylori-related gastritis and Helicobacter pylori infection in tongue coating as well as tongue images of traditional Chinese medicine [M.D. thesis]*.

[23] Wang X., Sun H., Zhang A., Sun W., Wang P., Wang Z. (2011). Potential role of metabolomics apporoaches in the area of traditional Chinese medicine: as pillars of the bridge between Chinese and Western medicine. *Journal of Pharmaceutical and Biomedical Analysis*.

[24] Cao H., Zhang A., Zhang H., Sun H., Wang X. (2015). The application of metabolomics in traditional Chinese medicine opens up a dialogue between Chinese and Western medicine. *Phytotherapy Research*.

[25] Sun Z., Zhao J., Qian P. (2013). Metabolic markers and microecological characteristics of tongue coating in patients with chronic gastritis. *BMC Complementary and Alternative Medicine*.

[26] Chen F., Xue J., Zhou L., Wu S., Chen Z. (2011). Identification of serum biomarkers of hepatocarcinoma through liquid chromatography/mass spectrometry-based metabonomic method. *Analytical and Bioanalytical Chemistry*.

[27] Leichtle A. B., Nuoffer J., Ceglarek U. (2012). Serum amino acid profiles and their alterations in colorectal cancer. *Metabolomics*.

[28] Wang J. L., Li Y. L. (1992). *She Zhen Yuan Jian*.

[29] Shi B., Xu H., Xie J. (2008). The significance of TCM tongue diagnosis in the treatment of chronic gastritis. *Forum on Traditional Chinese Medicine*.

[30] Li F., Zhao J., Qian P. (2012). Metabolite changes in the greasy tongue coating of patients with chronic gastritis. *Journal of Chinese Integrative Medicine*.

[31] Zhao Y., Gou X., Dai J. (2013). Differences in metabolites of different tongue coatings in patients with chronic hepatitis B. *Evidence-Based Complementary and Alternative Medicine*.

[32] Aoyama K., Watabe M., Nakaki T. (2008). Regulation of neuronal glutathione synthesis. *Journal of Pharmacological Sciences*.

[33] McNulty C. A., Dent J. C. (1987). Rapid identification of *Campylobacter pylori* (*C. pyloridis*) by preformed enzymes. *Journal of Clinical Microbiology*.

